# A novel expressed prostatic secretion (EPS)-urine metabolomic signature for the diagnosis of clinically significant prostate cancer

**DOI:** 10.20892/j.issn.2095-3941.2020.0617

**Published:** 2021-06-15

**Authors:** Denise Drago, Annapaola Andolfo, Ettore Mosca, Alessandro Orro, Luigi Nocera, Vito Cucchiara, Matteo Bellone, Francesco Montorsi, Alberto Briganti

**Affiliations:** 1 ProMeFa, Proteomics and Metabolomics Facility, Center for Omics Sciences (COSR), IRCCS San Raffaele Scientific Institute, Milan 20132, Italy; 2 Institute of Biomedical Technologies, National Research Council (CNR), Milan 20090, Italy; 3 Department of Urology and Division of Experimental Oncology, Urological Research Institute (URI), IRCCS San Raffaele Scientific Institute, Milan 20132, Italy; 4 Division of Immunology, Transplantation and Infectious Diseases, IRCCS San Raffaele Scientific Institute, Milan 20132, Italy

**Keywords:** Prostate, cancer, EPS-urine, metabolomics, prediction, diagnosis

## Abstract

**Objective:**

Significant efforts are currently being made to identify novel biomarkers for the diagnosis and risk stratification of prostate cancer (PCa). Metabolomics can be a very useful approach in biomarker discovery because metabolites are an important read-out of the disease when characterized in biological samples. We aimed to determine a metabolomic signature which can accurately distinguish men with clinically significant PCa from those affected by benign prostatic hyperplasia (BPH).

**Methods:**

We first performed untargeted metabolomics using ultrahigh-performance liquid chromatography tandem mass spectrometry on expressed prostatic secretion urine (EPS-urine) from 25 patients affected by BPH and 25 men with clinically significant PCa (defined as Gleason score ≥ 3 + 4). Diagnosis was histologically confirmed after surgical treatment. The EPS-urine metabolomic approach was then applied to a larger, prospective cohort of 92 consecutive patients undergoing multiparametric magnetic resonance imaging for clinical suspicion of PCa prior to biopsy.

**Results:**

We established a novel metabolomic signature capable of accurately distinguishing PCa from benign tissue. A metabolomic signature was associated with clinically significant PCa in all subgroups of the Prostate Imaging Reporting and Data System (PI-RADS) classification (100% and 89.13% of accuracy when the PI-RADS was in range of 1–2 and 4–5, respectively, and 87.50% in the more critical cases when the PI-RADS was 3).

**Conclusions:**

A combination of metabolites and clinical variables can effectively help in identifying PCa patients that might be overlooked by current imaging technologies. Metabolites from EPS-urine should help in defining the diagnostic pathway of PCa, thus improving PCa detection and decreasing the number of unnecessary prostate biopsies.

## Introduction

Although prostate-specific antigen (PSA) represents a mainstay for prostate cancer (PCa) diagnosis, PSA alone is associated with significant rates of false-negative and false-positive findings. Indeed, a large proportion of men diagnosed with PCa through PSA screening have an indolent disease not necessarily progressing into an aggressive cancer phenotype. Moreover, high PSA levels can be found in patients affected by benign prostatic hyperplasia (BPH) as well as prostatic infections. Therefore, improper and unjustified adoption of PSA screening might lead to the implementation of potentially unwarranted invasive procedures both for diagnosis and treatment.

In this context, PCa overdiagnosis and overtreatment is certainly fueled by the lack of reliable tools to discriminate accurately between men with clinically indolent PCa and those with more aggressive disease^1^. Currently, the gold standard for the diagnosis and staging of PCa is the histopathologic assessment of the core needle biopsy, which provides a measure of the extent of cancer in the examined tissue, along with information about the architectural aspects of cancer foci (e.g., loss of glandular structure as characterized by Gleason score). However, the accuracy of needle biopsy in detecting whole-gland abnormalities is limited by its untargeted nature and by the heterogeneity and multifocality of PCa. Recently, technological advances and a greater understanding of tumor biology have opened the way of a new era in PCa detection^2^. Multiparametric magnetic resonance imaging (mp-MRI) has shown high negative predictive value for the detection of clinically significant PCa (defined as Gleason score ≥ 3 + 4)^3^, and it is currently recommended for all patients with elevated PSA and clinical suspicion of PCa prior to biopsy (EAU guidelines: https://uroweb.org/guideline/prostate-cancer/). Despite high negative predictive value, mp-MRI does have limitations, such as the risk of missing clinically significant disease in approximately 15% of patients. This is mainly due to two factors: 1) inability of MRI to detect small-volume, high-risk foci of PCa; 2) inaccurate estimation of equivocal lesions (defined as PI-RADS 3 lesions).

The inability of clinicopathological investigations to accurately predict PCa aggressiveness clearly underscores the need to determine the potential prognostic usefulness of additional biomarkers able to distinguish indolent from aggressive PCa. In this context, metabolomics may represent a very useful approach to discover novel biomarkers, since metabolites are an important read-out of disease when present in biological samples such as tissues and body fluids. Moreover, prostate is known to exhibit a unique metabolite profile^4^. Specifically, metabolites in body fluids (e.g., urine, serum) as well as in radical prostatectomy tissues have been correlated with PCa aggressiveness and progression^5–11^. However, only few studies investigated the metabolic content of expressed prostatic secretion (EPS) and none of them implemented metabolomics into mp-MRI diagnostic pathways. EPS represent an attractive source of potential PCa biomarkers because these fluids bathe the tumor^12,13^. EPS is secreted by the prostate following a digital rectal prostate massage and can be collected in voided bladder after the procedure. It contains proteins and metabolites that are secreted/released from the prostate into the extracellular environment and that might reflect prostate “health status” much better than needle biopsy. Despite its importance and potential applications, a complete characterization of EPS-urine is not yet available. Specifically, although the proteome of EPS has already been found to be a rich source of biomarkers^14–17^, the metabolomics profile of EPS-urine has not been studied yet.

Here, we exploited EPS-urine and ultrahigh-performance liquid chromatography tandem mass spectrometry (UPLC/MS-MS) to identify novel biomarkers that distinguish BPH from PCa, thus proving a specific metabolomic signature of PCa. We also prospectively recognized the EPS-urine metabolomic signature in association with the mp-MRI in an additional cohort of patients who underwent mp-MRI at initial prostate biopsy for clinical suspicion of PCa.

## Materials and methods

### Study population

After we obtained institutional review board approval, we prospectively selected 50 consecutive patients, of whom 25 were affected by BPH and 25 were affected by PCa, scheduled for surgical treatment (i.e., transurethral resection or enucleation for BPH patients and robot-assisted radical prostatectomy for PCa patients). All men had EPS-urine collected on the day of surgery. After acquisition of the expressed patient consent, EPS-urine samples were collected upon bladder voiding. Subsequent prostatic massage was performed with three strokes per lobe during rectal examination of the prostate^12^. The clinical characteristics of the examined population are summarized in **Table 1**.

**Table 1:** Descriptive characteristics of the study population composed of 50 consecutive patients

Variable	Overall (*n* = 50)	BPH (*n* = 25, 50%)	cs-PCa (*n* = 25, 50%)	*P* value
Age (years)	67 [62.2–74.8]	71 [63–79]	67 [62–70]	0.07
BMI (kg/m^2^)	24.5 [24–25.9]	24.2 [23.5–25.2]	24.7 [24.1–26.1]	0.75
PSA level (ng/mL)	4.2 [2.9–6.9]	3.1 [1.6–4.4]	5.9 [4–8.5]	<0.001
Prostate volume (mL)	55 [40.8–77.2]	74 [46–99]	50 [38–56]	<0.05
GGG				
2	12 (24)	–	12 (48)	
3	9 (18)	–	9 (36)	
5	4 (8)	–	4 (16)	

BPH, benign prostatic hyperplasia; PCa, prostate cancer; BMI, body mass index; PSA, prostate-specific antigen; GGG, Gleason grade group. Values are presented as median [interquartile range] or *n* (%).

Following the first development and proof-of-concept phase, a second study was performed by prospectively collecting data from additional 92 two consecutive patients who underwent mp-MRI at our institution for clinical suspicion of PCa based on increased PSA levels and digital rectal examination. The clinical characteristics of the second examined population are summarized in **Table 2**.

**Table 2:** Descriptive characteristics of the study population composed of 92 patients

Variable	Overall (*n* = 92*)	No PCa (*n* = 37, 40.2%)	PCa (*n* = 55, 59.8%)	*P* value
Age (years)	67 [61.8–74.2]	64 [60–69]	71 [63–76]	<0.01
PSA level (ng/mL)	6.3 [4.3–9.9]	5.3 [4–7.9]	7.4 [4.8–10.4]	<0.05
Prostate volume (mL)	55 [40–70]	60 [45–73]	50 [38–70]	0.17
GGG				
1	13 (14.1)	0 (0)	13 (23.6)	
2	18 (19.6)	0 (0)	18 (32.7)	
3	13 (14.1)	0 (0)	13 (23.6)	
4	7 (7.6)	0 (0)	7 (12.7)	
5	4 (4.3)	0 (0)	4 (7.3)	
Clinical T stage				0.07
1	70 (76.1)	32 (86.5)	38 (69.1)	
2	19 (20.7)	4 (10.8)	15 (27.3)	
3	2 (2.2)	0 (0)	2 (3.6)	
PI-RADS score				<0.001
1	2 (2.2)	2 (5.4)	0 (0)	
2	9 (9.8)	7 (18.9)	2 (3.6)	
3	32 (34.8)	18 (48.6)	14 (25.5)	
4	29 (31.5)	8 (21.6)	21 (38.2)	
5	17 (18.5)	0 (0)	17 (30.9)	

BPH, benign prostatic hyperplasia; PCa, prostate cancer; PSA, prostate-specific antigen; GGG, Gleason grade group; PI-RADS, Prostate Imaging Reporting and Data System. Values are presented as median [interquartile range] or *n* (%). *Three of 92 patients had no PI-RADS value.

Written informed consent was provided by all the participants.

### Sample collection and preparation

Approximately 50 mL of EPS-urine were obtained from each patient and centrifuged at 1,280 g at 4 °C for 30 min to remove any possible cellular contamination. After collection, 10 mL of each sample were immediately aliquoted (1 mL each aliquot) and stored at −80 °C until LC/MS-MS analysis. Twenty-five PCa and 25 BPH EPS-urine samples were collected. The PCa and BPH EPS-urine samples were subdivided into 5 groups of 5 samples each. EPS-urine samples belonging to the same group were pooled together for the LC-MS/MS analysis. The analyses were performed in three technical replicates. Quality control (QC) samples, made up of all samples pooled together, were run within the queue every 5 samples to monitor the performance of the LC-MS/MS system over time.

Additional 92 patients, who were candidate for prostate biopsies for clinical suspicion of PCa but with either negative (PI-RADS 1–2) or equivocal (PI-RADS 3) lesions at mp-MRI or with a positive mp-MRI (PI-RADS 4–5) were also enrolled in the prospective study. The clinical characteristics of the second examined population are summarized in **Table 2**. Three out of 92 patients had no PI-RADS value. Their EPS-urine samples were collected as previously described. Every EPS-urine sample was analyzed by LC-MS/MS in three technical replicates. QC samples, made up of all samples pooled together, were run within the queue every 5 samples to monitor the performance of the LC-MS/MS system over time.

### LC-MS/MS

Metabolomic profiling of EPS-urine was performed using UPLC 1290 system (Agilent Technologies, Santa Clara, CA, USA) coupled with a TripleTOF 5600+ MS (Sciex, Framingham, MA, USA) equipped with an electrospray ionization source. Reverse-phase C18 columns (Waters ACQUITY UPLC HSS T3 C18 10 × 2.1 mm, 1.8 µm) and hydrophilic interaction liquid chromatography (Waters ACQUITY UPLC BEH amide 10 × 2.1 mm, 1.7 µm) were used to cover a wide range of metabolites based on their chemical properties. The chromatographic separation by reverse-phase C18 columns was performed according to Want et al.^18^ with some modifications. Briefly, 20 µL of EPS-urine were directly injected upon dilution 1:3 with solvent A (water, 0.1% formic acid) for reverse-phase C18 analysis. Metabolites were separated using a flow rate set at 0.6 mL/min and a gradient of solvent A and B (methanol, 0.1% formic acid). The gradient in both the positive and negative modes was 2% B for 1 min, up to 20% B in 3 min, up to 95% in 4 min, and at 95% B for 2 min. The column was set at 50 °C while the samples were kept at 4 °C. For BEH amide column analysis, the chromatographic separation was performed according to Paglia et al.^19^ with some modifications. Briefly, 5 µL of EPS-urine were directly injected upon dilution 1:2 with solvent A (ACN, 0.1% formic acid) for hydrophilic interaction liquid chromatography analysis. Metabolites were separated using a flow rate set at 0.4 mL/min and a gradient of solvent A and solvent B (water, 0.1% formic acid). The gradient, in both positive and negative modes, was in 7 min from 1 up to 70% B. The column was set at 40 °C while the samples were kept at 4 °C. The TripleTOF 5600+ system was used for data acquisition over a mass range of 50–500 *m*/*z*.

Automated calibration was performed using an external calibrant delivery system, which infused APCI-positive or APCI-negative calibration solution every 5 sample injections. A time-of-flight mass spectrometry (TOF MS) survey scan experiment with an information-dependent acquisition (IDA) experiment was set to monitor the 8 most intense candidate ions (accumulation time of 150 msec in TOF-MS and 50 msec in IDA experiment) with a collision energy of 35 ± 10 V, a declustering potential of 80 V, source temperature of 500 °C, and ion-spray voltage floating of 5500 V in high sensitivity mode. The method was applied both in positive and negative polarities, with appropriate corrections (collision energy, −35 ± 10 V; declustering potential, −80 V, ion-spray voltage floating, −4500 V).

### Data processing

All data were processed using MasterView™ software (Sciex) for metabolite identification with the Accurate Mass Metabolite Spectral Library (Sciex). For the first part of the study (EPS-urine metabolomics analysis; 25 BPH *vs.* 25 PCa patients), MarkerView™ software (Sciex) was used for simultaneous feature finding, alignment (retention time width: 1 min and 10 s). Significant metabolites for each experimental comparison were used for the principal component analysis (PCA). Since normalization is recommended to improve the differential profile between sample groups by detecting and decreasing unwanted variations arising from errors in the EPS withdrawal^20^, probabilistic quotient normalization (PQN)^21^ was applied. MultiExperiment Viewer version 4.9.0^22^ (freely downloadable) was used for hierarchical clustering heatmap of peak area of the significant differentially regulated metabolites among experimental conditions both in positive and negative polarities, applying Pearson correlation metric (*P* < 0.05). This analysis was performed on the mean values of the three technical replicates for each group of EPS-urine samples. Pathways analysis was also carried out on the PQN normalized data using web-free available MetaboAnalyst version 4.0^23^ (freely available). Mapped pathways were ranked according with their enrichment and topological analysis performed by the MetPa method implemented in MetaboAnalyst. The pathway impact is calculated as the sum of the importance measures of the matched metabolites normalized by the sum of the importance measures of all metabolites in each pathway.

For the prospective study on 92 patients, a batch correction step was also added in the data processing to avoid bias in the peak area calculation due to the temporal difference in the LC-MS/MS acquisition process. Pairs of features assigned to the same metabolite and having the most similar retention times were aggregated (aligned), and the mean retention time was defined as representative of the aggregated pair. The procedure was repeated for all pairs that had a difference of retention times of at most 1 min with an additional tolerance of 10 s. Within each batch, only features with at least 5 not null values were considered. The same aggregation scheme was first applied to each batch separately and then to the resulting features of the six batches. The obtained data were normalized through the PQN method^21^, using the median of each metabolite among the QC samples to define the reference profile. PQN was first applied within each batch and then between batches. The values of a metabolite in three technical replicates were averaged only if at least two values were available; otherwise, all three values were set to zero. Only features with non-zero values in most of the patients (70%) were used in differential abundance assessment by *t*-tests between healthy and prostate cancer groups both in positive and negative. Two comparisons were considered: all 92 patients and only the most critical group characterized by PI-RADS 3.

### Prediction model

A set of combinations of 1 to 6 metabolites plus 1 to 3 clinical variables were used to evaluate naive Bayes predictors using cross validation. For each classifier, several measures have been computed such as accuracy, true-positive, true-negative, false-positive, and false-negative rates. Then, the list has been sorted and ranked in order to extract the predictor that performs better in the three classes of interest (PI-RADS 1–2, PI-RADS 3, and PI-RADS 4–5), taking into account both the accuracy and the true-negative rate. Because of the limited size of the dataset, leave-one-out cross-validation was adopted: each sample was selected iteratively to evaluate the accuracy of the predictor trained with the remaining 91 samples. In this way, 92 validated predicted outputs were obtained, and relevant parameters were considered such as accuracy, true-positive rate, and false-negative rate were used to create a confusion matrix.

The naive Bayes is a prediction model in which all variables are assumed statistically independent so that a simplified product formula can be used to estimate the probability of each class. For example, for the healthy condition (*H*):

$$\begin{array}{l} P(H|x_{1},x_{2},\ldots )=\frac{P(x_{1},x_{2},\ldots |H)\cdot P(H)}{P(x_{1},x_{2,}\ldots )}\\ \;=P(x_{1}|H)\cdot P(x_{2}|H)\cdot \ldots \cdot \frac{P(H)}{P(x_{1},x_{2},\ldots )} \end{array}$$

where *x*_1_, *x*_2_, … are the variables (metabolite abundance and clinical variables). All probabilities in the right side of the equation were modeled as Gaussian curve with mean and standard deviation obtained from the training set.

Given a new sample characterized by variables *x*_1_, *x*_2_, …, the prediction is obtained with the previous formula by evaluating the probability that the sample is in the class “healthy” or “PCa” and considering the class associated with the maximum probability as output.

## Results

### EPS-urine metabolomic signature differentiates BPH from PCa patients

An untargeted metabolomic study was performed on EPS-urine obtained from 25 patients with clinically significant PCa (defined as biopsy Gleason score ≥ 3 + 4) and 25 men with BPH who were scheduled for surgical treatment. Polar and apolar metabolites were simultaneously extracted and profiled using two chromatographic columns (C18 and BEH amide).

**Figure 1:**
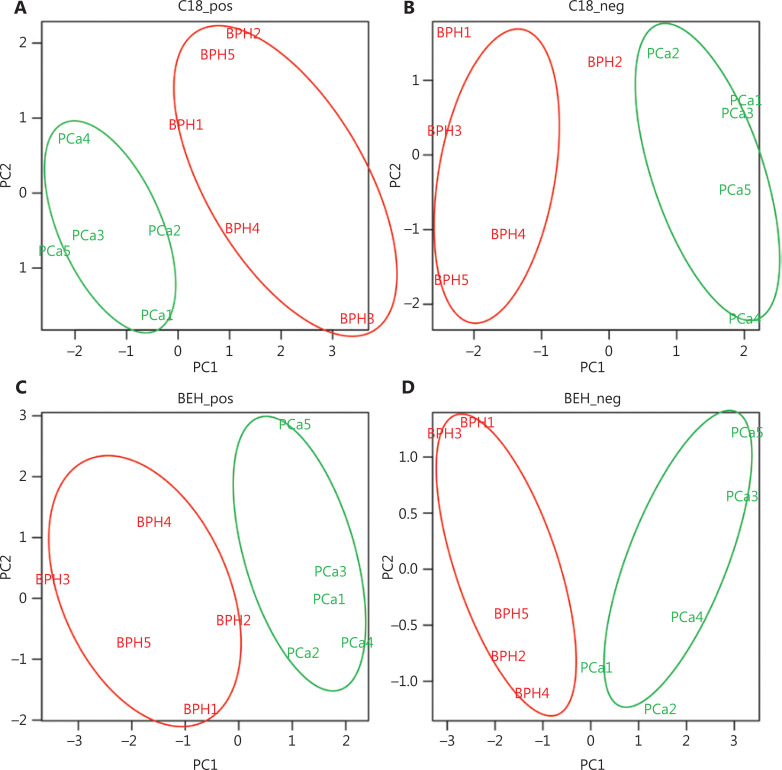
Untargeted metabolomics analysis of BPH and PCa patients (C18 chromatographic separation): unsupervised principal component analysis score for C18 and BEH amide column separation in positive (A, C) and negative (B, D) modes assessing the clustering of the BPH (red) and PCa (green) patients performed on identified metabolites upon probabilistic quotient normalization. BPH, benign prostatic hyperplasia; PCa, prostate cancer; BPH, benign prostatic hyperplasia.

**Figure 2:**
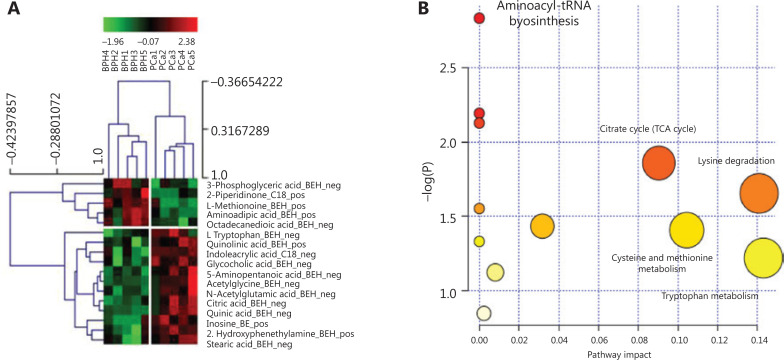
EPS metabolomic signature of BPH and PCa patients. (A) Seventeen significantly (*P* < 0.05) different metabolites between BPH and PCa patients. Chromatographic column (C18 or BEH) and mass spectrometry polarity (positive or negative) are indicated. (B) The most significant metabolic pathways for BPH and PCa are represented by bigger/red dots with higher −log *P* value. EPS, expressed prostatic secretion; BPH, benign prostatic hyperplasia; PCa, prostate cancer; pos, positive; neg, negative.

To define the metabolomic signature that could discriminate between the two groups of patients, we compared several normalization approaches: logarithmic, total area, protein content, and PQN. PCA plots for each normalization method are presented in **Supplementary Figure S1**. The PQN methodology was the most reliable, recommended by several studies^21^, and consistent with the other methods, as it revealed the presence of several metabolites differentially expressed between EPS-urine of patients with PCa and BPH. PCA on PQN normalized data clearly segregated BPH from PCa patients in separate clusters (**Figure 1**), thus indicating that BPH and PCa metabolic profiles were highly specific. More in details, the C18 column identified 55 metabolites in positive modality and 86 metabolites in negative modality. Using the BEH amide column, we also identified 79 metabolites in positive modality and 80 metabolites in negative modality (**Supplementary Table S1a– S1d**). By applying the *t*-test between PCa and BPH patients, we identified 17 metabolites that were significantly different (*P* < 0.05, out of 300 metabolites) in the two groups (**Supplementary Table S1e**), as also indicated by the hierarchical clustering (**Figure 2A**). The C18 column retrieved 2-piperidinone and indoleacrylic acid as differentially expressed metabolites, whereas 15 metabolites resulted differentially expressed using the BEH amide column (**Figure 2A**).

To identify the intracellular metabolic pathways that were mostly altered in PCa (*vs.* BPH), a metabolic pathway analysis (MetPA) was performed on differentially represented metabolites using MetaboAnalyst, a web-based tool for metabolomic data interpretation^23^ (**Figure 2B**). Metabolites belonging to the citrate cycle (TCA cycle), lysine degradation, cysteine and methionine metabolism, and Tryptophan metabolism were enriched in PCa and presented the highest impact value (i.e., pathway impact value calculated from pathway topology analysis) (**Supplementary Table S1f**).

### Prediction model based on mp-MRI patients

**Figure 3:**
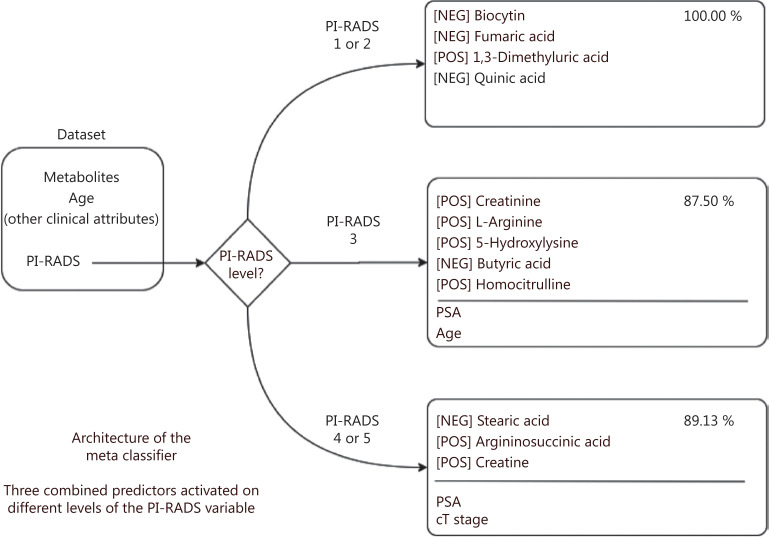
Structure of the meta classifier: the PI-RADS value is used to choose a different set of variables as input for the corresponding classifier. The metabolites for each PI-RADS level are indicated along with the polarity (positive or negative) for mass spectrometry acquisition. PI-RADS, Prostate Imaging Reporting and Data System; pos, positive; neg, negative.

The untargeted metabolomic approach developed in the first part of this work was then applied to prospectively investigate 92 patients undergoing mp-MRI for PCa diagnosis. Because the BEH amide column identified the vast majority of the significantly different metabolites in PCa/BPH patients (**Figure 1**), we used this column for further analyses. Similarly, taking into account the previous results on the comparison of several normalization methods, we only applied the PQN methodology. A total of 200 metabolites in positive polarity and 207 in negative polarity were thus identified upon PQN methodology (**Supplementary Table S2a and S2b**). To select for the most discriminating metabolites between PCa patients and control subjects, the metabolites with 70% of non-missing values (**Supplementary Table S2a and S2b**) were considered using either all patients (**Supplementary Table S2c and S2d**) or those classified as PI-RADS 3, the most problematic subgroup for clinical decisions (**Supplementary Table S2e and S2f**). We found a number of metabolites that changed between PCa and control subjects, and even if these changes were marginally significant when analyzing each metabolite independently, they could be predictive of PCa status when combined together. Therefore, we used 10 metabolites with the highest differences in each comparison (**Supplementary Table S3**) along with the most relevant clinical variables to develop a model to predict the presence of PCa (**Supplementary Table S4**). The different behaviors of the three sets of metabolites associated with the model suggest the definition of a meta classifier (MC) that activates the corresponding model depending on the known value of the PI-RADS. We therefore obtained an MC that includes three naive Bayes models, each one based on a specific set of variables optimized to obtain the highest accuracy in a PI-RADS subgroup (**Figure 3**). The theoretical accuracy of the MC of approximately 89.89% can be easily obtained with the average of the three accuracies weighted by the number of samples in each category, specifically 100%, 87.50%, and 89.13% for PI-RADS 1–2, PI-RADS 3, and PI-RADS 4–5, respectively. The distribution of the more predictive variables in each PI-RADS class highlights the complex relationship among metabolite levels, clinical attributes, and patient state (**Supplementary Figures S2–S4**).

Predictors were tested in three relevant subsets of samples according to mp-MRI findings: probably healthy (PI-RADS 1–2), difficult to diagnose (PI-RADS 3), and probably sick (PI-RADS 4–5) (**Supplementary Table S5**). As expected, predictive accuracy was higher when mp-MRI was positive (100% and 89.13% of accuracy when PI-RADS is in range of 1–2 and 4–5, respectively) than in more equivocal cases (87.50% when PI-RADS was 3) (**Supplementary Table S6**). However, for PI-RADS 3-classified patients, the predictive contribution of metabolite level and clinical variables was more pronounced as compared to the other groups (+ 31.25% for PI-RADS 3 against + 18.19% and + 6.52% for PI-RADS 1–2 and 4–5, respectively), when using only PI-RADS classification (**Supplementary Table S6**). The addition of clinical variables did not significantly improve the prediction above the use of PI-RADS only (**Supplementary Table S6**). On the contrary, the metabolite levels provided significant benefit in all cases (**Supplementary Table S6**). In addition, to compare our results with state-of-the-art methods in terms of diagnosis and PCa patient stratification, the metabolites included in the commercial Prostarix™ prostate cancer test (Metabolon Inc., Durham, NC) (alanine, glycine, glutamic acid and sarcosine) were used to train a naive Bayes model. The obtained results for the 92 patients are reported in **Supplementary Table S6**, showing high accuracy only in the PI-RADS 4–5 subgroup (**Supplementary Table S6**).

**Figure 4:**
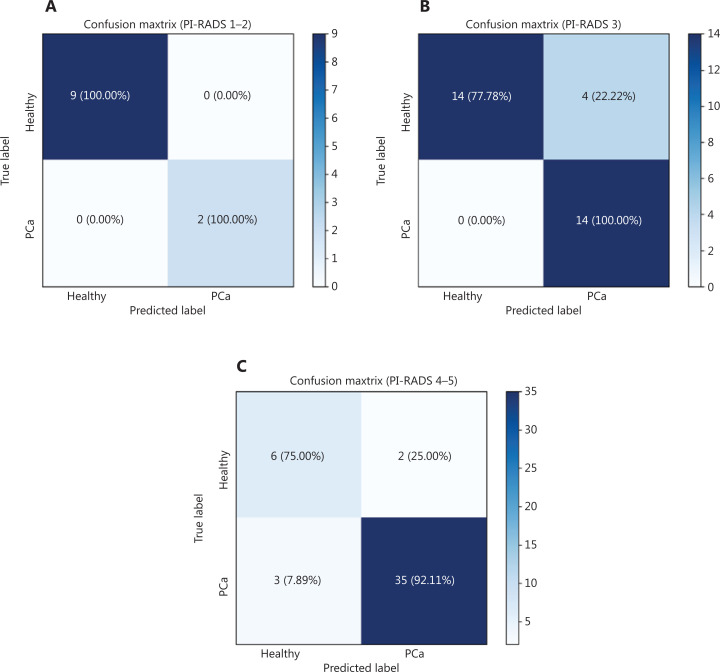
Confusion matrix: true-positive, true-negative, false-positive, and false-negative rates in the prediction model. (A) PI-RADS 1-2. (B) PI-RADS 3. (C) PI-RADS 4-5.

Analysis by confusion matrices (**Figure 4**) highlighted other important characteristics of the model, the most relevant being the sensitivity value of 100% in the PI-RADS 3 subgroup, with a considerably high specificity value of 77.78%. In the “gray zone” of the PI-RADS 3 subgroup consisting of 32 patients, 14 men were correctly predicted as healthy and 14 patients as affected by PCa and 4 patients not correctly assigned, with 87.50% of accuracy in the prediction (**Figure 4B**). Among 11 patients within the PI-RADS 1–2 group, 9 patients were correctly predicted as healthy and 2 patients as affected by PCa, with a sensitivity and specificity of virtually 100% (**Figure 4A**). Among 46 patients with PI-RADS 4–5, our model was able to correctly classify 6 patients as healthy and 35 patients as affected by PCa, showing a sensitivity of 92.11%, a specificity of 75%, and 89.31% of accuracy in the prediction (**Figure 4C**).

All together, these findings demonstrate the usefulness of measuring EPS-urine metabolites to improve the diagnostic accuracy of PCa patients and identify a metabolic signature that would be particularly important in classifying PI-RADS 3 patients.

## Discussion

Promising results from metabolomics urinary fingerprinting analyses suggest that there is more to be studied in the field of metabolomic biomarkers in EPS-urine. This is a highly novel and challenging field of investigation that brings an exciting new technology such as metabolomics in the PCa biomarker discovery field.

Diagnostic usefulness of metabolomic biomarkers in routine clinical practice has already been demonstrated^24^. Specifically, metabolites in body fluids (e.g., urine, serum) as well as in RP tissues have been correlated with PCa progression^5–11^. Recently, sarcosine, proline, kynurenine, uracil, and glycerol 3-phosphate were found in high concentrations in metastatic prostate cancer urine samples^25,26^. This finding, however, was not confirmed by Jentzmik et al.^27,28^, who did not observe any significant correlation between sarcosine levels in post-DRE urine or tissues and prostate cancer aggressiveness. Cao et al.^29^ reported only a modest correlation between sarcosine levels and PCa progression, and the prognostic value of sarcosine was found to be inferior to the more robust prostate cancer gene 3 (PCA3) and PSA biomarker^30^. Therefore, the diagnostic and prognostic impact of sarcosine in prostate cancer progression remains controversial. As for sarcosine, more consistent studies are needed for other metabolites. In fact, it remains elusive whether metabolomics findings could aid in the diagnosis of PCa and in patient stratification based on tumor aggressiveness.

Only few studies used EPS-urine, and none of them implemented metabolomics into mp-MRI diagnostic pathways that are now the standard of care for PCa diagnosis. Since EPS bathe the tumor, it is an attractive source of potential PCa biomarkers, containing informative proteins and metabolites that might better reflect prostate “health status” than needle biopsy. Although the proteome of EPS has already been found to be a rich source of biomarkers^14–17^, the metabolomics profile of EPS has not been studied yet.

Our untargeted metabolomic approach on EPS-urine provided metabolomic signatures that clearly discriminated between PCa and BPH patients and more accurately classified patients undergoing mp-MRI for PCa diagnosis. Our experimental design did not comprise healthy donors because our goal was to propose a diagnostic tool that can enhance the performance of mp-MRI, applied only to those cases of PCa suspicion. Our approach provides an additional level of accuracy, beside PSA level and other clinical parameters, to calculate the individual risk of clinically significant PCa. By using the novel metabolomics signature, we could find the way to improve the performance characteristics of mp-MRI and compare its performance *vs.* that of commercially available kit such as Prostarix™.

For example, in the PI-RADS 4–5 case, our best predictor was composed of three metabolites (stearic acid, argininosuccinic acid, and creatine) and two clinical variables (PSA and cT-stage). Notably, stearic acid was significantly up-represented in PCa compared with BPH patients in the first part of this work, in line with literature, as recently reviewed by Giunchi et al.^31^ They also concluded that greater *de novo* fatty acids synthesis is a hallmark of PCa^31^. Thus, our metabolomics results not only confirmed the predictive value of stearic acid in EPS-urine from PCa patients but also showed that stearic acid is one of the metabolites predicting the more aggressive PI-RADS 4–5 case, thus indicating the specificity of the prediction.

The other two metabolites selected for PI-RADS 4–5 (i.e., argininosuccinic acid and creatine) are part of the arginine metabolism^32^. Arginine is a semi-essential amino acid, whose endogenous synthesis, under physiological conditions, is sufficient to meet the body requirements. However, during infancy, growth, pregnancy, and illness, such as infections and cancer, arginine is synthesized in multiple tissues by the arginine–citrulline cycle^33^. Indeed, arginine is a major metabolic hub for the synthesis of multiple metabolites, such as NO, polyamines, proline, and creatine, all of which are essential for cell survival and proliferation^34–36^. Besides NOS, the other two enzymes that function in the arginine–citrulline cycle are argininosuccinate synthase 1 (ASS1) and argininosuccinate lyase (ASL). ASS1 is a cytosolic enzyme that catalyzes the formation of argininosuccinate from citrulline and aspartate, with ATP being broken down into AMP and pyrophosphate during the reaction. Subsequently, ASL promotes the cleavage of argininosuccinate to arginine and fumarate. ASS1 is overexpressed in various human cancers^37^, but the cancer-promoting mechanisms fostered by ASS1, and their clinical implications, remain unclear. It is possible that high levels of ASS1, which can result in high levels of argininosuccinate, support tumor proliferation and aggressiveness by increasing the supply of arginine for NO production.

Five metabolites (creatinine, l-arginine, 5-hydroxylysine, butyric acid, and homocitrulline) and two clinical variables (PSA and age) were the best predictors in the PI-RADS 3 subgroup. Although l-arginine and homocitrulline belong to the arginine metabolism, butyrate can induce growth inhibition and apoptosis in numerous cancers, including prostate cancer^38^.

Finally, in the case of PI-RADS 1–2, our best predictor contains four metabolites (biocytin, fumaric acid, 1,3-dimetyluric acid, and quinic acid). Notably, tryptophan, quinolinic acid, and quinic acid were also found to be significantly overrepresented in EPS-urine from PCa compared with BPH patients, thus indicating a possible relevance of tryptophan metabolism also in the context of prostate cancer, in addition to its demonstrated role in colon^39^ and breast cancer^40^. Quinolinic acid is an essential precursor for *de novo* NAD+ synthesis. Under normal physiological conditions, the production of picolinic acid and quinolinic acid is at equilibrium at the end of the kynurenine pathway of tryptophan metabolism. However, during chronic activation, the kynurenine metabolism is diverted toward quinolinic acid production, and hence NAD+ biosynthesis, which may promote cellular growth and contribute to immune escape^40^.

## Conclusions

Our metabolic predictors associated with mp-MRI are expected to better guide physicians in their clinical decision-making process and also to have significant impact on the National Health System. Indeed, our novel metabolomic signature would decrease the number of unnecessary biopsies in men with negative/equivocal mp-MRI. Additionally, based on the identified metabolic signature, patients at low risk of multifocal, clinically significant PCa might undergo a decreased number of cores at biopsy. These patients would be candidate for mp-MRI-guided targeted biopsy only. This, in turn, would translate into significant reduction in costs and in complications associated with more extensive biopsy sampling.

The main limitations of our study are the small size of the cohort (overfitting in predictive models) and intrinsic technical difficulties in metabolomics data analysis (e.g., peak alignment, data normalization, missing values). Therefore, a further validation study on a bigger set of patients will clarify to which extent our model can be generalized. Moreover, targeted analysis for the selected panel of metabolites will be one of our future goals to determine the metabolite absolute quantification in EPS-urine in order to define a metabolic diagnostic score needed for a correct patient stratification. So far, our model has been assessed by cross-validation and has performed better than other metabolomics-based diagnosis methods, such as Prostarix™. It is not surprising that our model performed better than Prostarix™, since it was tailored on these patients; nevertheless, it includes a set of metabolites that are also present in this commercial diagnostic tool.

Our approach is also in line with the emerging trend toward the shift from trans-rectal prostate biopsy to “liquid biopsy”. Although direct analysis of tumor tissues may potentially provide access to greater concentrations of tumor-specific metabolites, their extreme heterogeneity would likely result in unsatisfactory yields of tissue-derived tumor-specific metabolites. On the contrary, EPS-urine is obtainable in a straightforward, noninvasive fashion. Thus, it is a clinically attractive biofluid that can be used to routinely screen for prostate biomarkers in combination with mp-MRI, eventually offering important aid to clinicians in the clinical decision making process of PCa diagnosis.
