# Supplementary material for: A novel expressed prostatic secretion (EPS)-urine metabolomic signature for the diagnosis of clinically significant prostate cancer
Source: Cancer Biol Med. 2021 Jun 15;18(2):604–15. doi: 10.20892/j.issn.2095-3941.2020.0617 (PMC8185872; doi:10.20892/j.issn.2095-3941.2020.0617)
Supplement: Supplementary file 1 [file cbm-18-604-s001.pdf]

Supplementary materials

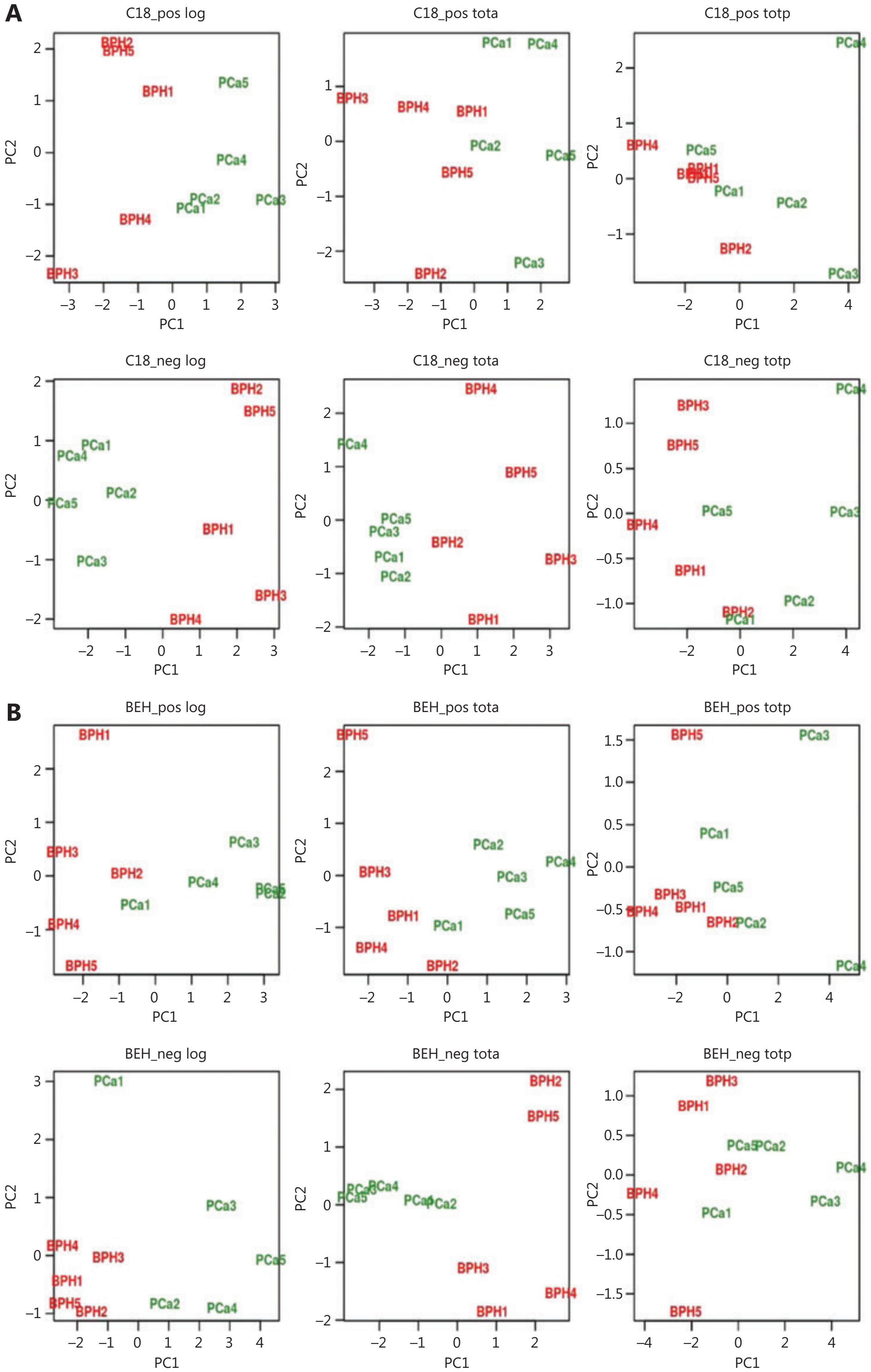

Figure S1 Untargeted metabolomics analysis of BPH and PCa patients: unsupervised principal component analysis score for C18 (A) and BEH amide (B) column separation in positive (pos) and negative (neg) mode assessing the clustering of the BPH (red) and PCa (green) patients performed on identified metabolites upon logarithmic (log), total area (tota), protein content (totp) normalization approach. BPH, benign prostatic hyperplasia; PCa, prostate cancer; pos, positive; neg, negative; log, logarithmic; tota, total area; totp, protein content.

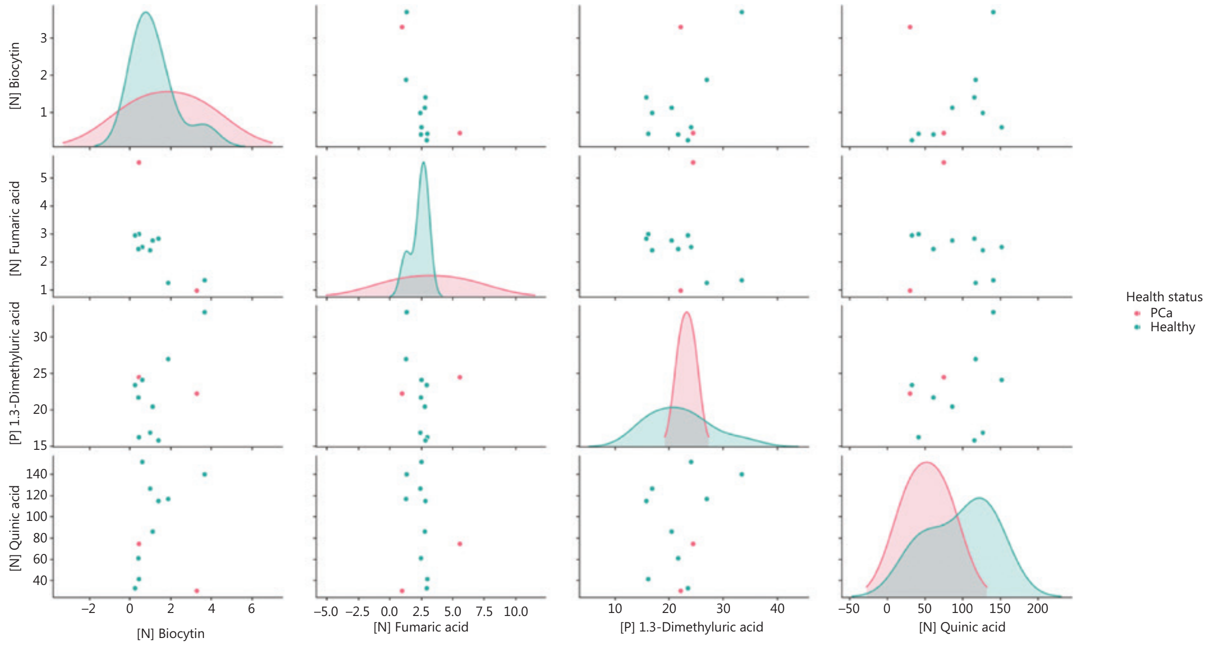

Figure S2 Data distribution of the more predictive variables for Prostate Imaging Reporting and Data System 1–2. Scatter plots and distributions of all pairs of predictive variables.

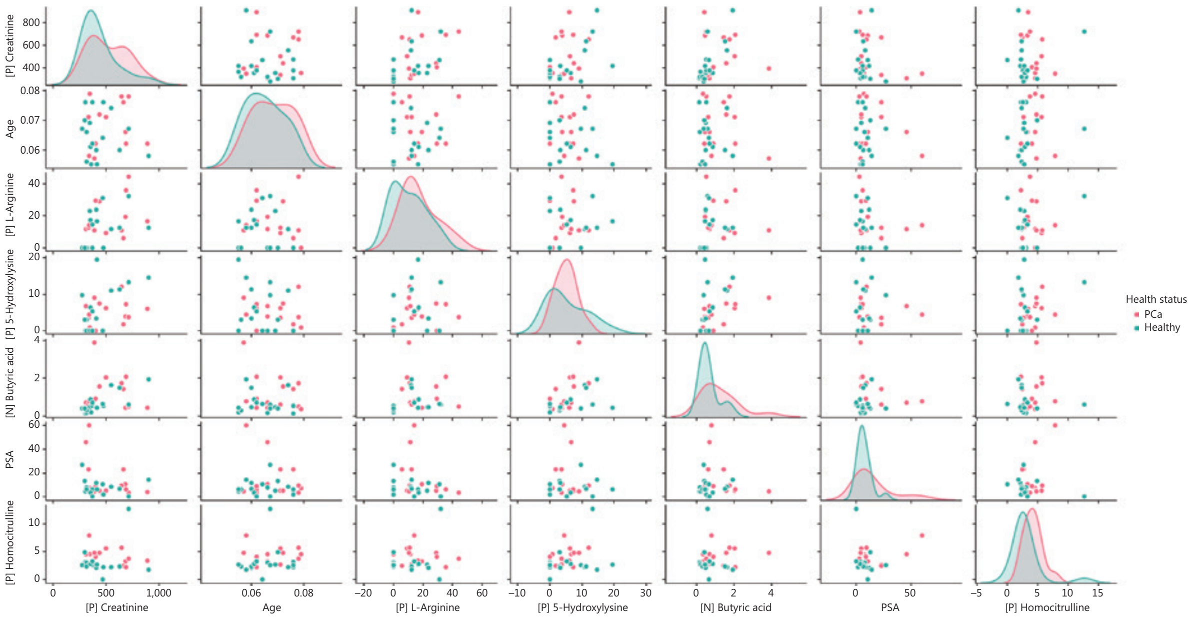

Figure S3 Data distribution of the more predictive variables for Prostate Imaging Reporting and Data System 3. Scatter plots and distributions of all pairs of predictive variables.

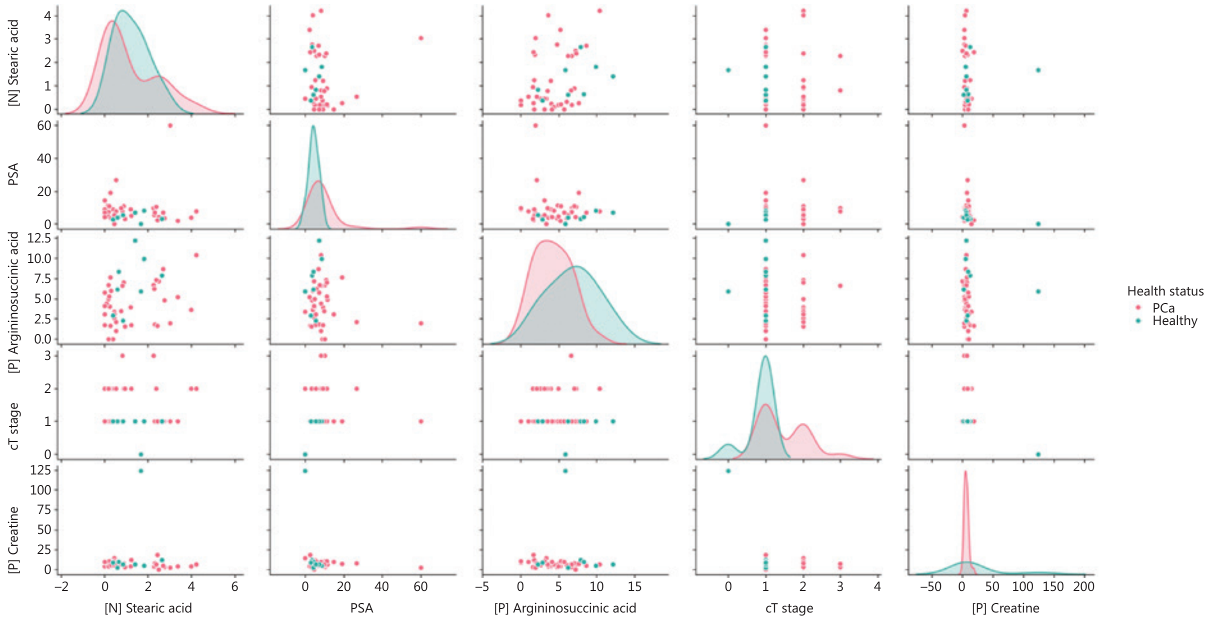

Figure S4 Data distribution of the more predictive variables for Prostate Imaging Reporting and Data System 4–5. Scatter plots and distributions of all pairs of predictive variables.

Table S3 Metabolites used for developing the predictive model. Upon selection of 10 metabolites with the most marked significant differences in each comparison (all patients and only Prostate Imaging Reporting and Data System 3, positive and negative polarities), the top 31 metabolites were considered as candidate variables for developing a predictive model of patient state

POS/NEG	Metabolites
POS	1,3-Dimethyluric acid
POS	2-Pyrrolidinone
POS	4-Aminohippuric acid
POS	5-Hydroxylysine
POS	7-Methylguanine
POS	Argininosuccinic acid
POS	Creatine
POS	Creatinine
POS	Glycyl-L-leucine
POS	Hippuric acid
POS	Homocitrulline
POS	L-Arginine
POS	L-Cystathionine
POS	L-Serine
POS	*N*-acetyl-L-methionine
NEG	2-Hydroxy-2-methylbutyric acid
NEG	Acetylcysteine
NEG	α-Aspartyl-lysine
NEG	Biocytin
NEG	Butyric acid
NEG	Canrenone
NEG	Creatinine
NEG	Fumaric acid
NEG	Glyceric acid
NEG	Histamine
NEG	Isovalerylglycine
NEG	L-Aspartyl-L-phenylalanine
NEG	Malonic acid
NEG	Quinic acid
NEG	Stearic acid
NEG	Xanthosine

Table S4 Clinical variables and the relative description

Clinical variables	Description
PI-RADS	Prostate Imaging Reporting and Data System
Age	Age of the patient
cT stage	Clinical T category
Prostate volume	Volume of the prostate
PSA	Serum prostate-specific antigen

Table S5 PI-RADS classification with the relative number of 92 patients analyzed in the metabolomics approach

PI-RADS	Description	No. samples per group
1–2	Probably healthy	11
3	Difficult to diagnose	32
4-5	Probably sick	46
NA	Not assessed	3
Total		92

PI-RADs, Prostate Imaging Reporting and Data System; NA, not assessed.

Table S6 Percentage of accuracy in the three subsets of samples (PI-RADS 1–2, 3, and 4–5). The relative percentage of accuracy for the prediction model trained using our meta classifier, only PI-RADS classification, clinical variables including PI-RADS classification, or Prostarix™ kit metabolites

PI-RADS	Accuracy (%)			
	MC	PI-RADS only	Clinical variables including PI-RADS	Prostarix metabolites™
1–2	100	81.81	72.72	18.18
3	87.5	56.25	65.63	46.88
4-5	89.13	82.61	76.09	82.61
All	89.89	72.83	71.74	60.87

PI-RADS, Prostate Imaging Reporting and Data System; MC, meta classifier.
